# p38 MAPK activation through B7-H3-mediated DUSP10 repression promotes chemoresistance

**DOI:** 10.1038/s41598-019-42303-w

**Published:** 2019-04-09

**Authors:** Karine Flem-Karlsen, Christina Tekle, Tove Øyjord, Vivi A. Flørenes, Gunhild M. Mælandsmo, Øystein Fodstad, Caroline E. Nunes-Xavier

**Affiliations:** 1 Department of Tumor Biology, Institute for Cancer Research, Oslo University Hospital Radiumhospitalet, Oslo, Norway; 2 Institute for Clinical Medicine, Faculty of Medicine, University of Oslo, Oslo, Norway; 3 Department of Pathology, Oslo University Hospital Radiumhospitalet, Oslo, Norway; 4 Department of Medical Biology, Faculty of Health Sciences, UiT/The Arctic University of Norway, Tromsø, Norway

## Abstract

Immunoregulatory protein B7-H3 is involved in the oncogenic and metastatic potential of cancer cells, as well as in drug resistance. Resistance to conventional chemotherapy is an important aspect of melanoma treatment, and a better understanding of how B7-H3 enhances drug resistance may lead to the development of more effective therapies. We investigated the *in vitro* and *in vivo* sensitivity of chemotherapeutic agents dacarbazine (DTIC) and cisplatin in sensitive and drug resistant melanoma cells with knockdown expression of B7-H3. We found that knockdown of B7-H3 increased *in vitro* and *in vivo* sensitivity of melanoma cells to the chemotherapeutic agents dacarbazine (DTIC) and cisplatin, in parallel with a decrease in p38 MAPK phosphorylation. Importantly, in B7-H3 knockdown cells we observed an increase in the expression of dual-specific MAP kinase phosphatase (MKP) DUSP10, a MKP known to dephosphorylate and inactivate p38 MAPK. DUSP10 knockdown by siRNA resulted in a reversion of the increased DTIC-sensitivity seen in B7-H3 knockdown cells. Our findings highlight the potential therapeutic benefit of combining chemotherapy with B7-H3 inhibition, and indicate that B7-H3 mediated chemoresistance in melanoma cells is driven through a mechanism involving DUSP10-mediated inactivation of p38 MAPK.

## Introduction

B7-H3, a member of the B7 family of immune checkpoint proteins, is upregulated in many different cancer types^1^, and B7-H3 targeted therapy is currently being tested in several clinical trials^2^. B7-H3 has been found to favor tumor growth, cell proliferation, migration, invasion, and drug resistance^3–5^, although many aspects regarding its oncogenic potential are still unknown. For instance, B7-H3 has been involved in various signal transduction pathways, including the JAK/STAT, PI3K/Akt, and the mitogen activated protein kinase (MAPK) Raf/MEK/ERK pathways^2^, but their relation with chemoresistance is not fully understood.

The MAPK pathways regulate various cellular processes, such as proliferation, differentiation, apoptosis and stress responses, and include four major pathways, as defined by their MAPK effector: ERK1/2, ERK5, JNKs and p38s MAPK^6^. The p38 MAPK pathway is mainly activated by stress signals such as UV light, osmotic shock and cytokines^7^. When activated, the p38 MAPK pathway can phosphorylate a wide range of proteins. This include activating phosphorylation of various transcription factors that may, amongst many physiological processes, lead to the maintenance of a tumor aggressive phenotype and/or resistance to chemotherapy^8^.

The MAPKs are activated by phosphorylation by upstream kinases and inactivated by dephosphorylation of a group of dual specificity phosphatases called MAP kinase phosphatases (MKPs). MKPs include 10 active enzymes that show different specificity towards subgroups of MAPKs and have different localization patterns which permits a tight regulation, spatially and temporally, of the MAPK signaling. The MKPs can be divided into three groups: 1) the nuclear MKPs DUSP1, DUSP4, DUSP2, and DUSP5; 2) the cytoplasmic, ERK1/2-specific MKPs DUSP6, DUSP9, and DUSP7; and 3) the stress-activated, p38/JNK-specific MKPs: DUSP8, DUSP10, and DUSP16^9^.

Melanomas have classically been treated with chemotherapy, including DNA alkylating, platinum-based, and microtubule-interacting agents^10^. However, low response rates, high toxicity and resistance are commonly found^11^. In the past years, therapies with small-molecule inhibitors or antibodies targeting immune checkpoint proteins or BRAF have become more prominent^12^, but treatment resistance is frequent^13^. Despite the recent advances in melanoma treatment, dacarbazine (DTIC) chemotherapy is still being widely used even though its response rate as a single agent is only 10–20%^14,15^. Thus, improvement in melanoma therapy is highly needed. DTIC treatment combined with targeted therapy may improve the overall response, and is a promising way forward for treatment of metastatic melanoma^16^.

Inhibiting expression of B7-H3 has been found to increase cell and tumor sensitivity to various chemotherapeutic agents^4,17–21^. In this study, we investigated the mechanism behind B7-H3 mediated resistance to DTIC and cisplatin, using melanoma as a model. We identified modulation of p38 MAPK activation by DUSP10 as a novel mechanism of B7-H3-mediated chemoresistance.

## Results

### Reduced B7-H3 expression increases *in vitro* and *in vivo* sensitivity of melanoma cells to chemotherapy

**Figure 1:**
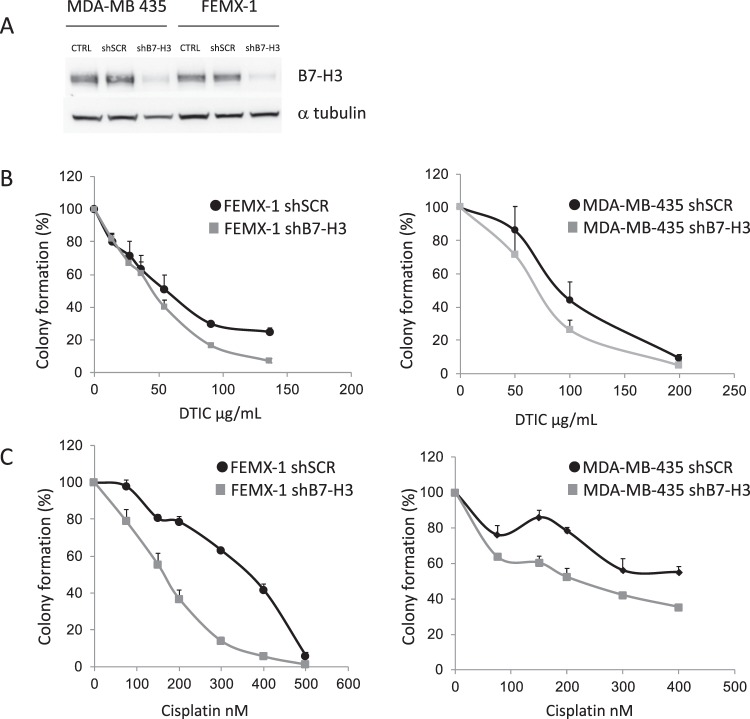
Melanoma cells with decreased B7-H3 expression have increased sensitivity to dacarbazine (DTIC) and cisplatin chemotherapy. (**A**) Immunoblot verifying the lentiviral knockdown of B7-H3 in MDA-MB-435 and FEMX-I cells. In cells were B7-H3 expression was decreased (short hairpin B7-H3, shB7-H3), the ability to form colonies in response to (**B**) DTIC and (**C**) Cisplatin chemotherapy treatment was reduced compared to control short hairpin scramble (shSCR) cells. Results show the average of three independent experiments ± SEM.

**Figure 2:**
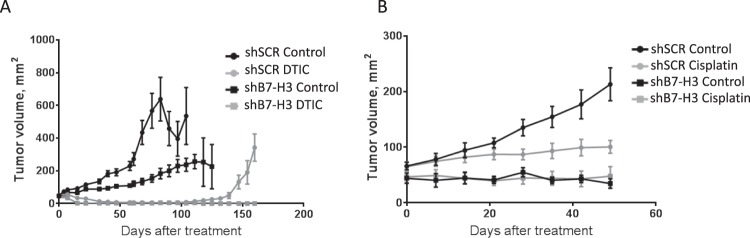
Tumor volume is reduced *in vivo* in shB7-H3 cells upon chemotherapy treatment. Mice were injected subcutaneously with 5 × 10^6^ MDA-MB-435 melanoma control cells (shSCR) and cells having diminished B7-H3 expression (shB7-H3). Upon B7-H3 knockdown, the relative tumor volume was reduced compared to control cells in response to (**A**) Dacarbazine (DTIC) (n = 20–22 in each group) and (**B**) Cisplatin (n = 5–6 in each group) chemotherapy treatment. Results show the relative tumor volume of the tumors ± SEM.

We have previously observed in *in vitro* proliferation assays that cells with decreased expression of B7-H3 display increased sensitivity to DTIC and small anti-cancer drugs, including molecular inhibitors^4^. However, the mechanism by which B7-H3 induces resistance to therapy is still unknown. To determine if the B7-H3 associated drug resistance might involve effects asserted by stress signaling, we treated melanoma cells with two chemotherapeutic agents, DTIC and cisplatin. The melanoma cell lines FEMX-I and MDA-MB-435 had stably reduced B7-H3 expression by short hairpin RNA (shRNA), as previously described (Fig. 1A; and in reference^5^). Diminished B7-H3 protein expression reduced the colony-formation ability of the cells upon treatment with both DTIC and cisplatin (Fig. 1B,C). This effect was also observed *in vivo* utilizing nude mice. Subcutaneous injection of cells with knocked down B7-H3 expression led to decreased relative tumor volume compared to control cells (Fig. 2A,B), and was further decreased when the mice were subjected to DTIC treatment (Fig. 2A). Of the DTIC treated mice 6/10 animals had regrowth at 100 days, while tumors in mice injected with B7-H3 knockdown cells showed regrowth in only 1/11 animals (Fig. 2A). Thus, melanoma cells with low expression of B7-H3 are more sensitive to DTIC and cisplatin chemotherapy.

### Chemotherapy resistance is abrogated in cells with reduced expression of B7-H3

**Figure 3:**
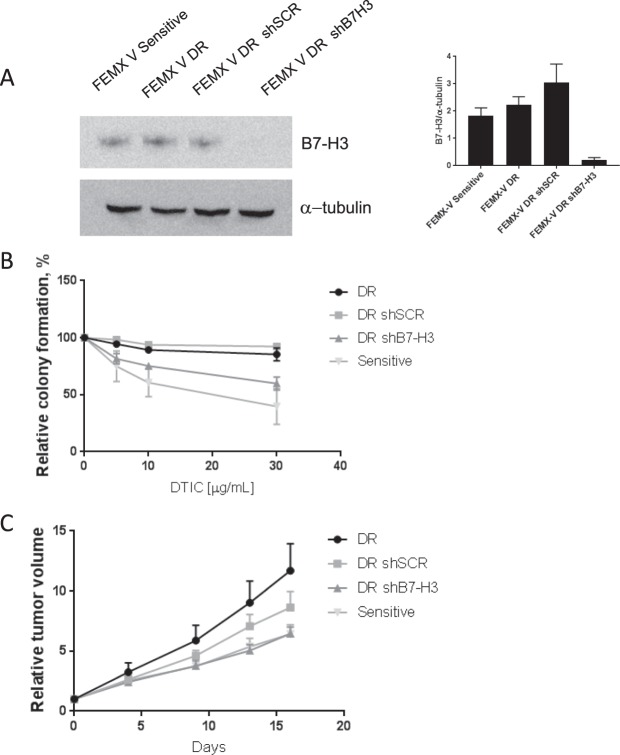
Reduced B7-H3 levels abolish dacarbazine (DTIC) resistance in DTIC resistant cells. FEMX-V cells with sensitivity (FEMX-V sensitive) or induced resistance to DTIC (FEMX-V DR), where knocked down for B7-H3. (**A**) Representative immunoblot of B7-H3 and α-tubulin expression in the four FEMX-V cell variants. (**B**) When these cells were subjected to DTIC treatment, little effect of DTIC was seen in the ability of drug resistant DR and DR shSCR cells to form colonies. FEMX-V DR shB7-H3 cells, however, displayed a reduced capacity to form colonies. Results show the average of two independent experiments ± SEM. (**C**) This effect was also seen *in vivo*, where FEMX-V shB7-H3 cells and FEMX-V sensitive cells displayed reduced relative tumor growth ± SEM compared to FEMX-V DR and FEMX-V DR shSCR cells (n = 7–8 in each group).

Interestingly, we observed 1.228-fold (SD ± 0.136265, p-value 0.004) higher B7-H3 expression in FEMX-V DTIC resistant (DR) cell line as compared to the sensitive parental cell line (Fig. 3A). Next, we investigated if reducing B7-H3 expression could increase sensitivity to chemotherapy in cells with induced drug resistance. To this end, we used the FEMX-V DTIC resistant (DR) cell line stably transduced with shRNA to reduce the B7-H3 expression as previously described (Fig. 3A). Upon DTIC treatment, the ability of FEMX-V shB7-H3 DR cells to form colonies was reduced to the level of FEMX-V sensitive control shSCR cells (Fig. 3B). Similarly, in xenograft experiments (Fig. 3C) the tumor growth curves were similar for mice injected with FEMX-V shB7-H3 DR cells to that of mice injected with FEMX-V sensitive cells. These data reinforce the notion that reducing B7-H3 expression independently abrogates the DTIC resistance of melanoma cells.

### DUSP10 expression is increased in shB7-H3 cells, and correlates with lower p-p38 MAPK levels

**Figure 4:**
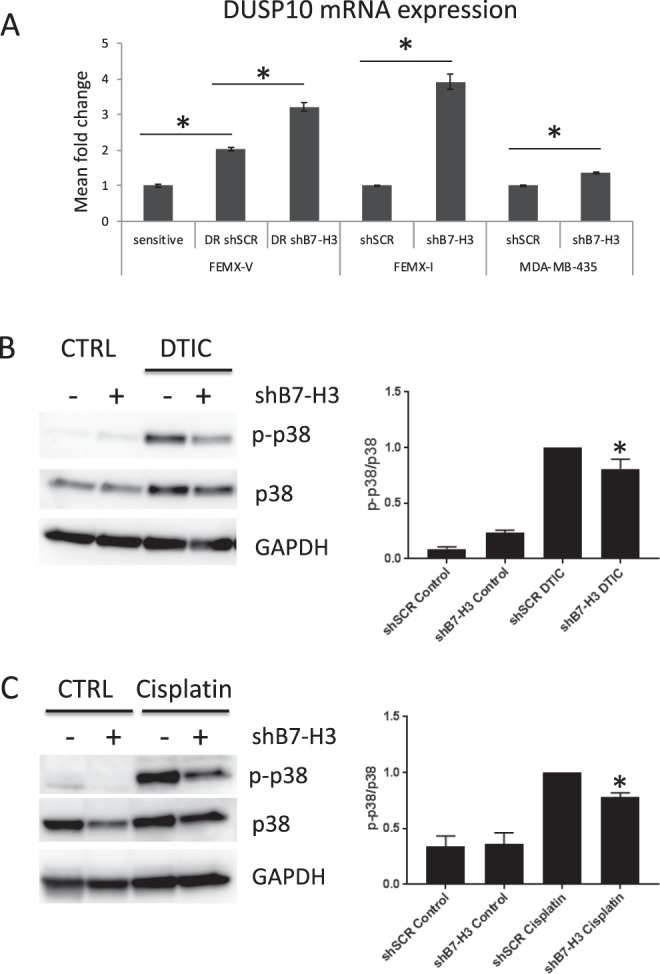
DUSP10 is induced in shB7-H3 cells and correlates with lower activation of p38 MAPK signaling by dacarbazine (DTIC) and cisplatin. (**A**) Mean fold change in gene expression of DUSP10 by qPCR of FEMX-V sensitive and DTIC resistant (DR), FEMX-I and MDA-MB-435 control (shSCR) and shB7-H3 cells. Representative immunoblot of phospho-p38 (p-p38), p38, and GAPDH levels in FEMX-I shSCR and shB7-H3 melanoma cells treated with: (**B**) 5 μg/mL DTIC or (**C**) 10 μg/mL cisplatin treatment. Right panels in B and C, average of three independent immunoblot values of p-p38 levels divided by p38 levels ± SEM.

In attempts to reveal the molecular basis of the involvement of B7-H3 in the drug resistance of FEMX-V DR cells, we performed a comparative DNA microarray gene expression analysis on FEMX-V DR control (shSCR) and shB7-H3 cells. Top differentially expressed genes with log2 value ±1 and p-value less than 0.05 are listed in Supplementary Table 1. Interestingly, one of the up-regulated genes was the dual-specific phosphatase DUSP10 (log2 Ratio = 1.052, p-value = 0.000124284). DUSP10 is a MAP kinase phosphatase known to dephosphorylate and negatively regulate p38 MAPK^9^. Up-regulation of DUSP10 mRNA expression was validated by qPCR (Fig. 4A). Additionally, we observed up-regulation of DUSP10 in FEMX-I shB7-H3 cells, and to a less extent in MDA-MB-435, shB7-H3 cells (Fig. 4A). To test the involvement of DUSP10 in regulating p38 MAPK activation, we analyzed p38 MAPK activation status in shSCR and shB7-H3 FEMX-I cells treated with DTIC or cisplatin. Both drugs induced p38 MAPK activation. Notably, this activation was lower in shB7-H3 knockdown cells compared to control cells treated with DTIC or cisplatin (Fig. 4B,C), suggesting that B7-H3 protein expression is involved in p38 MAPK activation.

### Increased drug sensitivity in shB7-H3 cells is reversed upon siRNA-induced down-regulation of DUSP10

**Figure 5:**
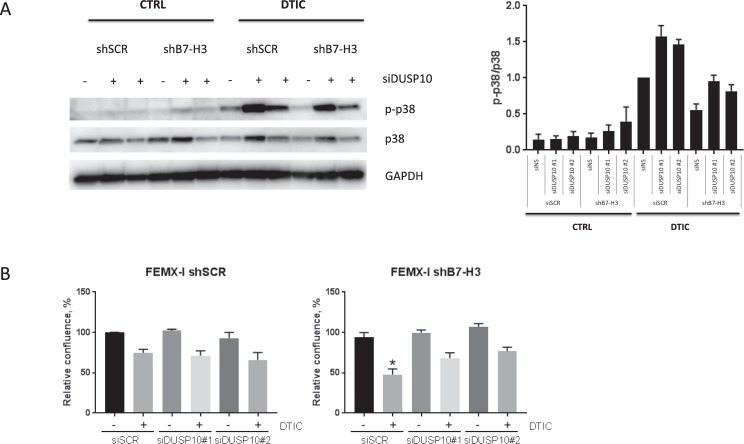
Reduced DUSP10 expression decreases chemosensitivity in shB7-H3 cells. (**A**) Left panel; Immunoblot analysis of phospho-p38 (p-p38), p38, and GAPDH expression upon DTIC treatment and right panel; average of three independent immunoblot values of p-p38 levels divided by p38 levels ± SEM in FEMX-I shSCR and shB7-H3 cells upon DUSP10 knockdown by siRNA. (**B**) Average proliferation of three independent experiments ± SEM of FEMX-I shSCR and shB7-H3 cells with DUSP10 knockdown and DTIC treatment as measured by the Incucyte FLR imaging system 72 h after treatment.

To analyze if DUSP10 levels affected the sensitivity to chemotherapy, we knocked down DUSP10 by siRNAs in shSCR and shB7-H3 FEMX-I cells. We observed a lower DTIC-induced activation of p38 MAPK in FEMX-I shB7-H3 cells compared to the shSCR cells, in consistence with higher DUSP10 expression (Figs 4A and 5A). Upon DUSP10 silencing, p38 MAPK activation was increased in both shSCR and shB7-H3 cells treated with DTIC. Moreover, the increased DTIC and cisplatin drug sensitivity achieved by B7-H3 knockdown was abrogated upon DUSP10 silencing (Fig. 5A,B and Supplementary Fig. 2). These results suggest that the increased chemosensitivity displayed by shB7-H3 cells is mediated through increased expression of DUSP10.

## Discussion

B7-H3 expression is associated with tumor progression and epigenetic regulatory activity in cutaneous melanoma^22^. B7-H3 expression in melanoma cells is also associated with sensitivity to various anti-cancer agents^4^, here including cisplatin. The mechanisms by which B7-H3 promotes drug resistance are largely unknown, although various pathways, such as JAK/Stat and PI3K/mTOR have been proposed to be involved^20,21^. In this study, we have identified the p38 MAPK pathway as a major effector of B7-H3-mediated resistance to chemotherapy and unveiled a novel B7-H3-associated regulation of p38 MAPK activation in melanoma cells. This regulation is mediated, at least in part, through the downregulation of the MAP kinase phosphatase DUSP10. Whether this B7-H3-DUSP10-p38 regulatory axis could be operative in other tumor types requires further studies. In this regard, p38 MAPK inactivation has been also observed in breast cancer cells upon knockdown of the B7-protein B7-H1/PD-L1^23^.

**Figure 6:**
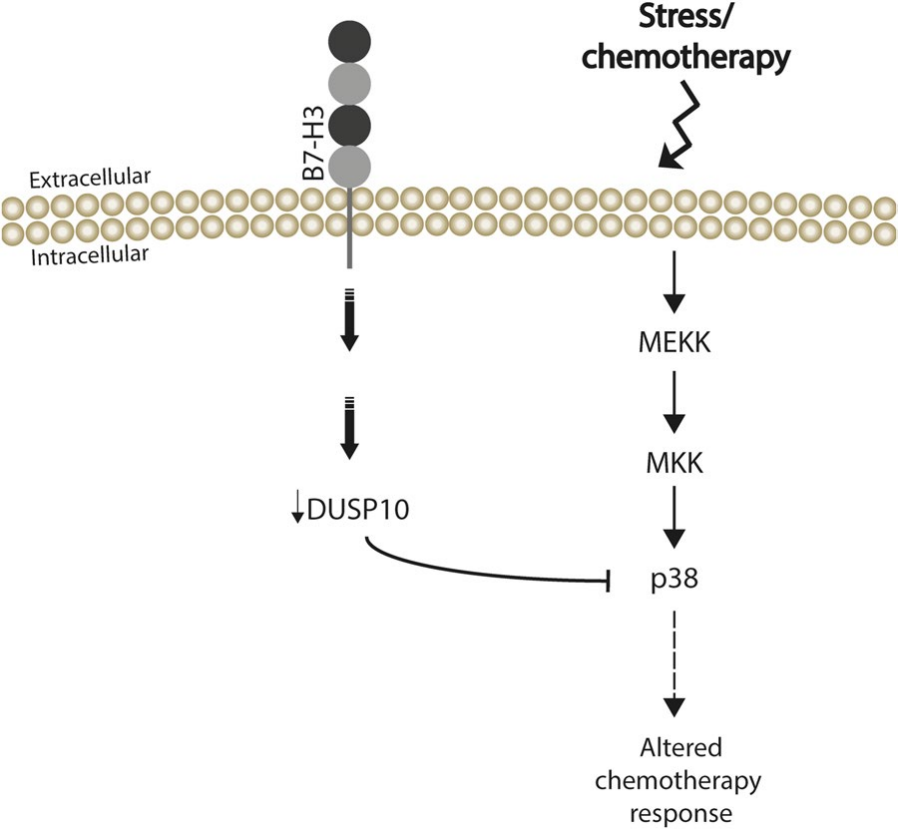
Schematic figure of how B7-H3 affects p38 MAPK signaling through modulation of DUSP10. In response to DTIC treatment, cells expressing B7-H3 have a lower level of DUSP10, which results in higher activation of p38 MAPK and resistance to chemotherapy. In cells with reduced B7-H3 levels, DUSP10 levels are higher and thus, p38 MAPK activation is lower, which leads to chemotherapy sensitivity. Upon reduction of DUSP10 levels, this sensitivity is eliminated.

Our results suggest that B7-H3 suppresses the expression of DUSP10 at the mRNA level, which in turn leads to higher p38 MAPK activation and increases tumor cell resistance to chemotherapy (Fig. 6). Thus, in our model, pharmacological inhibition of p38 MAPK would be beneficial and increase chemosensitivity of melanoma cells. It would be of interest to identify candidate DUSP10 transcription factors potentially repressed by B7-H3. DUSP10 is a stress-activated, JNK/p38-specific MKP widely expressed, reported to be involved in cancer progression and in the regulation of immune response^9^. Overexpression of DUSP10 in human colorectal cancer (CRC) cells resulted in reduced tumor formation in immune deficient mice, and high DUSP10 expression was associated with better survival in CRC patients^24^. Our *in vitro* and *in vivo* results also suggest an anti-oncogenic role for DUSP10 in melanoma. In addition, our findings support the existence of a B7-H3-DUSP10-p38 axis important for cell proliferation which is independent of the immune system.

Interestingly, up-regulation of DUSP10 in prostate cancer cells correlated with inactivation of p38 MAPK and decreased production of the inflammatory cytokine IL-6^25^, and DUSP10 was found to down-regulate the release of cytokines (IL-6 and TNF) by regulating p38 MAPK pathway in macrophages^26^. We have previously found B7-H3 expression to correlate with IL-8 secretion in melanoma cells^5^. Whether this phenomenon is mediated through DUSP10 needs further investigation.

In melanoma, activation of p38 MAPK has been associated with various cellular functions, including suppression of anti-tumor immune responses^27^, and resistance to chemotherapy^28^, and p38 MAPK inhibition has been found to suppress inflammation-induced metastasis^29^. Here, we observed activation of p38 MAPK in melanoma cells treated with either DTIC or cisplatin, indicating that these drugs affect cell proliferation through similar pathways. Inhibition of p38 MAPK is getting increased attention as a promising therapeutic approach in cancer^30^. Relevant to this, there are currently many clinical trials with various p38 MAPK inhibitors either alone or in combination with other chemotherapeutic agents in different types of cancers^31,32^. p38 MAPK inhibition increased the sensitivity to cisplatin in colorectal cancer^33^, and to taxanes in breast cancer cells^34^. It would be interesting to test whether these mechanisms could be dependent on B7-H3 expression.

Our findings support the idea that inhibiting B7-H3 may be a promising therapeutic concept in combination with chemotherapy. However, our *in vitro* and mouse xenograft models have limitations, and only reflect the B7-H3 tumor-intrinsic role. As B7-H3 is an immune checkpoint protein that prevents T cell activation, its inhibition could also affect immune function in the melanoma tumor microenvironment. Additional studies in immune competent mouse models would be necessary to assess the role of B7-H3 as an immune checkpoint protein and the potential of B7-H3 inhibition to promote both the anti-tumor immune response and sensitivity to chemotherapy in melanoma. Moreover, p38 MAPK signaling has been shown to induce the proliferation of regulatory T cells, thus dampening the immune response^35^. As B7-H3 expression correlates with the number of regulatory T cells^36^ and is known to exhibit a co-inhibitory signal on the immune system^37^, it would be interesting to investigate the activation of p38 MAPK by B7-H3 expression in immune competent models.

## Materials and Methods

### Cell cultures and silencing

FEMX-I and FEMX-V cell lines were established from a metastatic lymph node harvested from a melanoma patient operated at The Norwegian Radium Hospital^38^. FEMX-1 cells corresponds to the first generation of brain metastasis, and FEMX-V cells corresponds to the fifth generation, established by disaggregation, single cell formation and intraveneous re-injection in nude mice of the harvested cells as previously described^39^. MDA-MB-435 was acquired from ATCC. FEMX-V DTIC drug resistant (FEMX-V DR) cell lines were described previously^40^. B7-H3 knockdown in FEMX-V DR cells were made as reported for FEMX-I and MDA-MD-435 cells^5^, using HuSH 29mer shRNA constructs against B7-H3 (shB7-H3; sequence shRNA‐2, 5′-TCGTGTGCTGGAGAAAGATCAAACAGAGC‐3′) and control plasmid pRS nontarget TR30003 (shSCR; sequence 5′‐GCACTACCAGAGCTAACTCAGATAGTACT‐3′) (both from Origene Technologies). All cells were grown in RPMI-1640 (Sigma Aldrich) with addition of 10% fetal bovine serum and 2 mM L-glutamine. DUSP10 knockdown was performed by transfection of specific siRNAs using Lipofectamine 3000 (Thermo Fisher) following manufacturer’s protocol. DUSP10 siRNAs (siDUSP10 #1, SI03119998; siDUSP10 #2, SI03118178) were from Qiagen (Thermo Scientific), and siNS (non-specific) siRNA (J-003104-13) was from Dharmacon. The final concentration of Lipofectamin3000 and siRNAs were 2 μl/mL and 50 nM, respectively. DUSP10 knockdown was verified 72 h post-transfection at mRNA level by RT-qPCR, as described below.

### RNA isolation, DNA microarray and qPCR

The RNA was prepared for microarray analysis using the Illumina™ TotalPrep™ RNA amplification kit (Thermo Scientific) according to manufacturer’s protocol. The concentration of the samples was measured using NanoDrop spectrophotometer (Thermo Scientific) and the quality of the finished cRNA was assessed using the Bioanalyzer (Agilent Genomics). 1.5 µg biotin labeled cRNA was hybridized onto Illumina Human-6 Expression BeadChips (Illumina) using the Whole-Genome Gene Expression Direct Hybridization Assay (Illumina) according to manufacturer’s protocol. After scanning, the results were quality checked in Ilumina BeadStudio, and raw data were quantile normalized in log2 scale. The DNA microarray analyses were performed at the Genomics core facility, Oslo University Hospital (OUH), Norway. Total RNA was isolated for RT followed by qPCR using QuantiTect Primers (Qiagen) for DUSP10 and HPRT as a housekeeping gene as described previously in reference^41^.

### Reagents, immunoblot and antibodies

Decarbazine (DTIC) (Lipomed GmbH) and cisplatin (Accord Healthcare) were used at indicated concentrations, during the indicated times. Dimethyl sulfoxide (DMSO) was used as a control. Whole cell protein extracts were prepared by total cell lysis and immunoblot was performed as described previously in reference^4^. Antibodies used for Western blotting were: B7-H3 (1:1000, AF1027, R&D), phosoho-p38 (#9211), p38 (#8690), GAPDH (#5174) (1:1000, Cell Signaling) and α-tubulin (1:50000, CP06, Millipore). Protein concentrations from total cell lysates were measured using Pierce® BCA Protein Assay Kit (Thermo Scientific). Immunoblot expression levels were quantified using ImageJ. Uncropped blots are provided in the Supplementary Information.

### Cell proliferation and colony formation

To assess cell proliferation, 5000 cells/well were seeded on 96-well plates and the cell confluence was measured every three hours by the IncuCyte FLR or IncuCyte Zoom imaging microscopes (Essen Biosciences). The cells were treated with indicated drugs at indicated concentrations 21 h post-plating and were scanned for 72 h after adding the drugs. DMSO was added to control cells. For colony formation assays, 500 or 1000 cells/well were seeded on 6-well plates in media containing DMSO or indicated drugs. The cells were treated with drugs for 48 h, and plates were processed after 7 days. Colonies were counted after they were fixed with methanol and stained with 0.05% crystal violet.

### *In vivo* studies

The *in vivo* studies were performed using female nude athymic (fox1nu) mice bred at the Department of Comparative Medicine, Institute for Cancer Research, OUH Radiumhospitalet. When the animals were 6–8 weeks of age, 5 × 10^6^ cells were injected subcutaneously into both flanks of the nude mice. The treatment was initiated when the tumors were between 5–6 mm in diameter and consisted of a single treatment of 250 mg/kg DTIC or 10 mg/kg Cisplatin administered intravenously. Solvent was administered for control mice. Tumors were measured twice a week and the tumor volume was calculated by the formula 0.5 × length × width^2^. The data is presented as the average ± standard error of the mean (SEM) of three independent experiments. All animals were kept according to regulations of the Norwegian Animal Welfare Act and the experiments were approved by the Norwegian Animal Research Authority and conducted according to the FELASA guidelines (FOTS application number 1748 and 2499).

### Statistical analysis

Data shows average values ± SEM for the average of three representative experiments and *in vivo* experiments. All experiments were performed in technical and biological triplicates, if not otherwise specified. Data was analyzed by Graphpad Prism 7.0 (Graphpad Software), where significance was calculated using two-tailed students t-test. P values of less than 0.05 were considered significant and were marked with an asterisk.

## Conclusions

By using melanoma cells resistant to DTIC, we found that knocking down B7-H3 in these cells abolished the acquired resistance. These findings support the idea that inhibiting B7-H3 may be a promising therapeutic concept in combination with chemotherapy. Furthermore, we observed a parallel upregulation of the mRNA levels of the dual-specific MAPK phosphatase DUSP10 in the B7-H3 knockdown cells. Consistently, lower p38 MAPK activation upon chemotherapy was observed in cells with reduced B7-H3 expression in parallel with increased sensitivity. Moreover, the increased sensitivity of B7-H3 knockdown cells was abolished by DUSP10 knockdown by siRNA. Taken together, we have discovered a novel mechanism that contributes to B7-H3-mediated drug resistance through attenuating DUSP10 expression thereby activating p38 MAPK in melanoma cells.

## Supplementary information

Supplementary table 1

Supplementary information
