# Supplementary material for: p38 MAPK activation through B7-H3-mediated DUSP10 repression promotes chemoresistance
Source: Sci Rep. 2019 Apr 9;9:5839. doi: 10.1038/s41598-019-42303-w (PMC6456585; doi:10.1038/s41598-019-42303-w)
Supplement: Supplementary file 2 — Supplementary information [file 41598_2019_42303_MOESM2_ESM.pdf]

Supplementary information file for manuscript

Title: p38 MAPK activation through B7-H3-mediated DUSP10 repression promotes chemoresistance

Author list:
Karine Flem-Karlsen, Christina Tekle, Tove Øyjord, Vivi A. Flørenes, Gunhild M. Mælandsmo, Øystein Fodstad and Caroline E. Nunes-Xavier

Supplementary figure legends
Supplementary figures
Uncropped Western Blots
Figure 1A
Figure 3A
Figure 4B
Figure 4C
Figure 5C

Supplementary figure legends

Figure S1) qPCR analysis of DUSP10 expression in FEMX-I cells.

qPCR verifying DUSP10 knocked down in FEMX-I shSCR and shB7-H3 cells using two different siRNAs (siDUSP10 #1 and siDUSP #2).

Figure S2) Reduced DUSP10 expression decreases cisplatin chemosensitivity in shB7-H3 cells.

Average proliferation of three independent experiment ± SEM of FEMX-I shSCR and shB7-H3 cells with DUSP10 knockdown and indicated concentrations of cisplatin treatment as measured by the Incucyte FLR imaging system 72 h after treatment.

Supplementary Figure 1

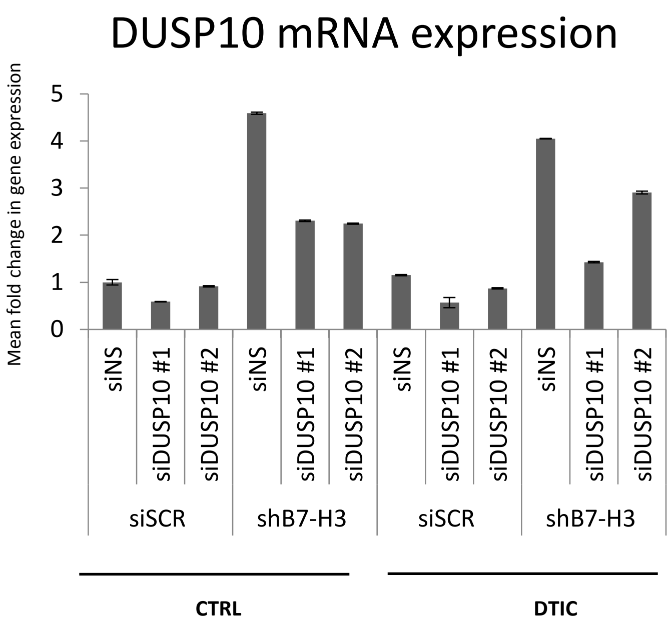

Supplementary Figure 2

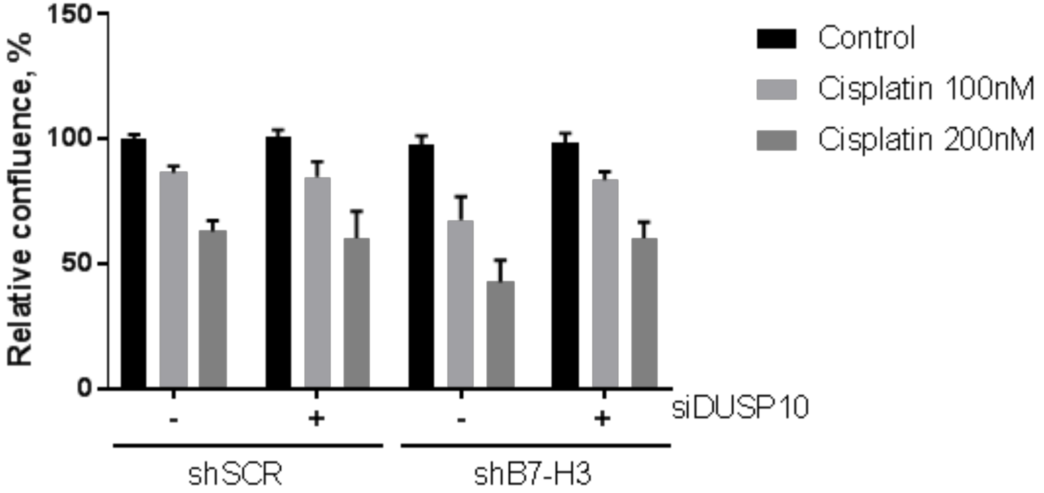

FIGURE 1A

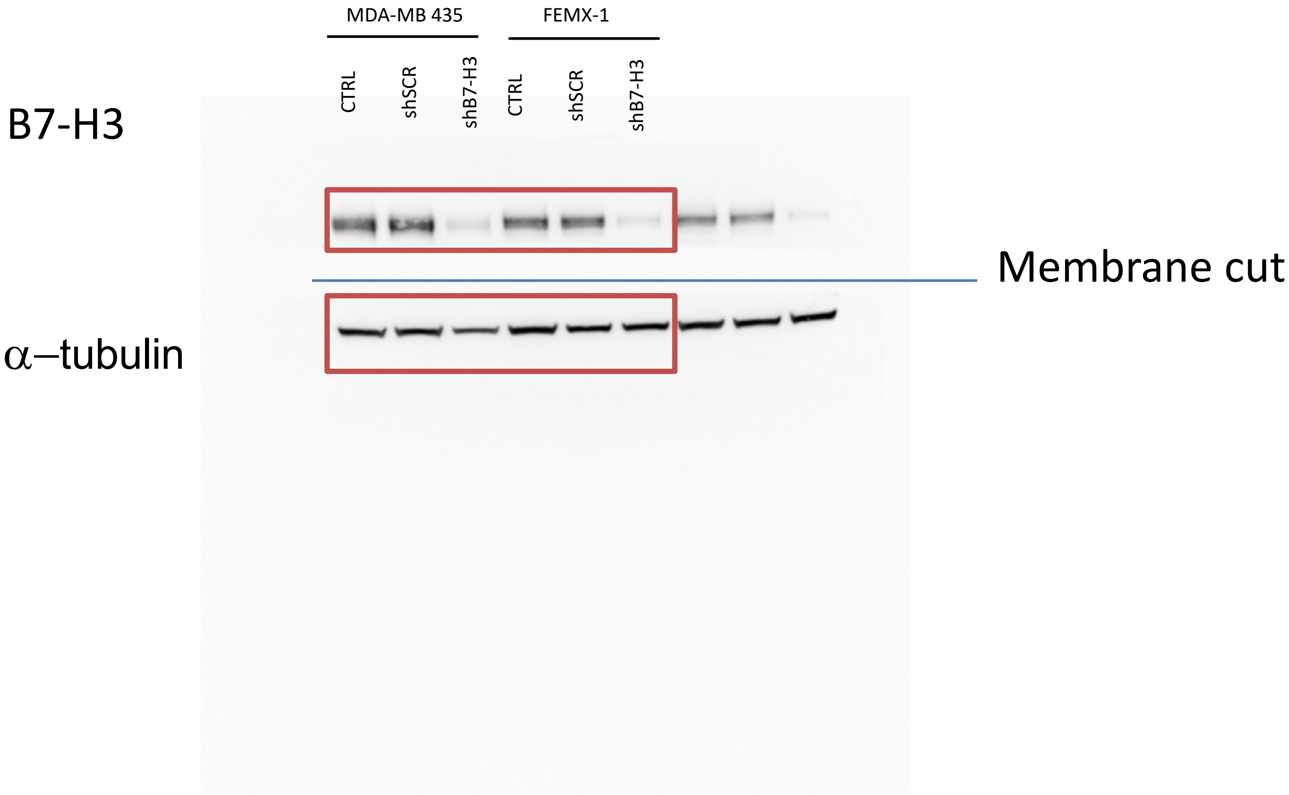
MDA-MB 435
FEMX-1
CTRL
shSCR
shB7-H3
CTRL
shSCR
shB7-H3
B7-H3
Membrane cut
α–tubulin

FIGURE 3A

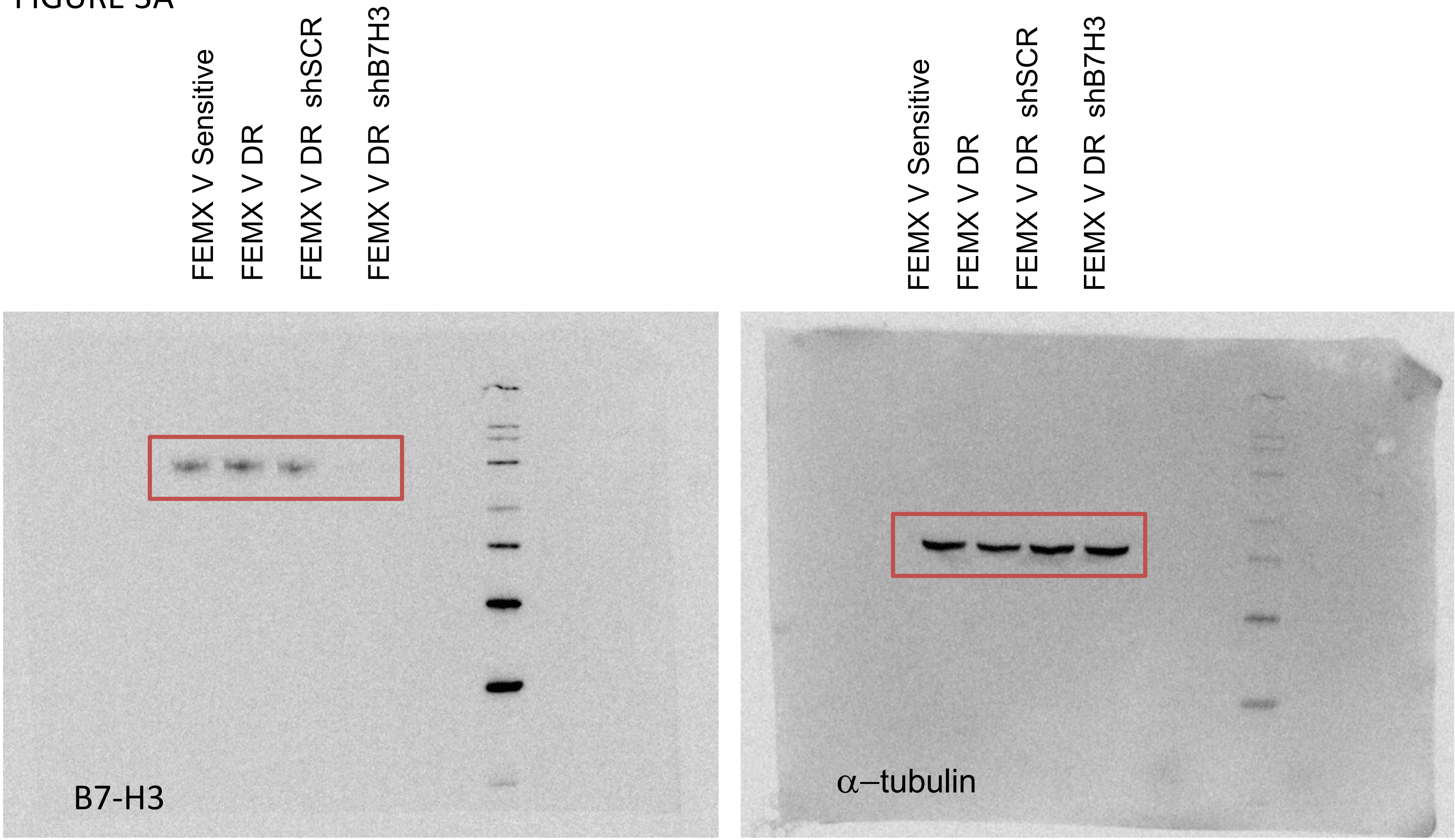
FEMX V Sensitive
FEMX V DR
FEMX V DR shSCR
FEMX V DR shB7H3
B7-H3
FEMX V Sensitive
FEMX V DR
FEMX V DR shSCR
FEMX V DR shB7H3
α–tubulin

FIGURE 4B

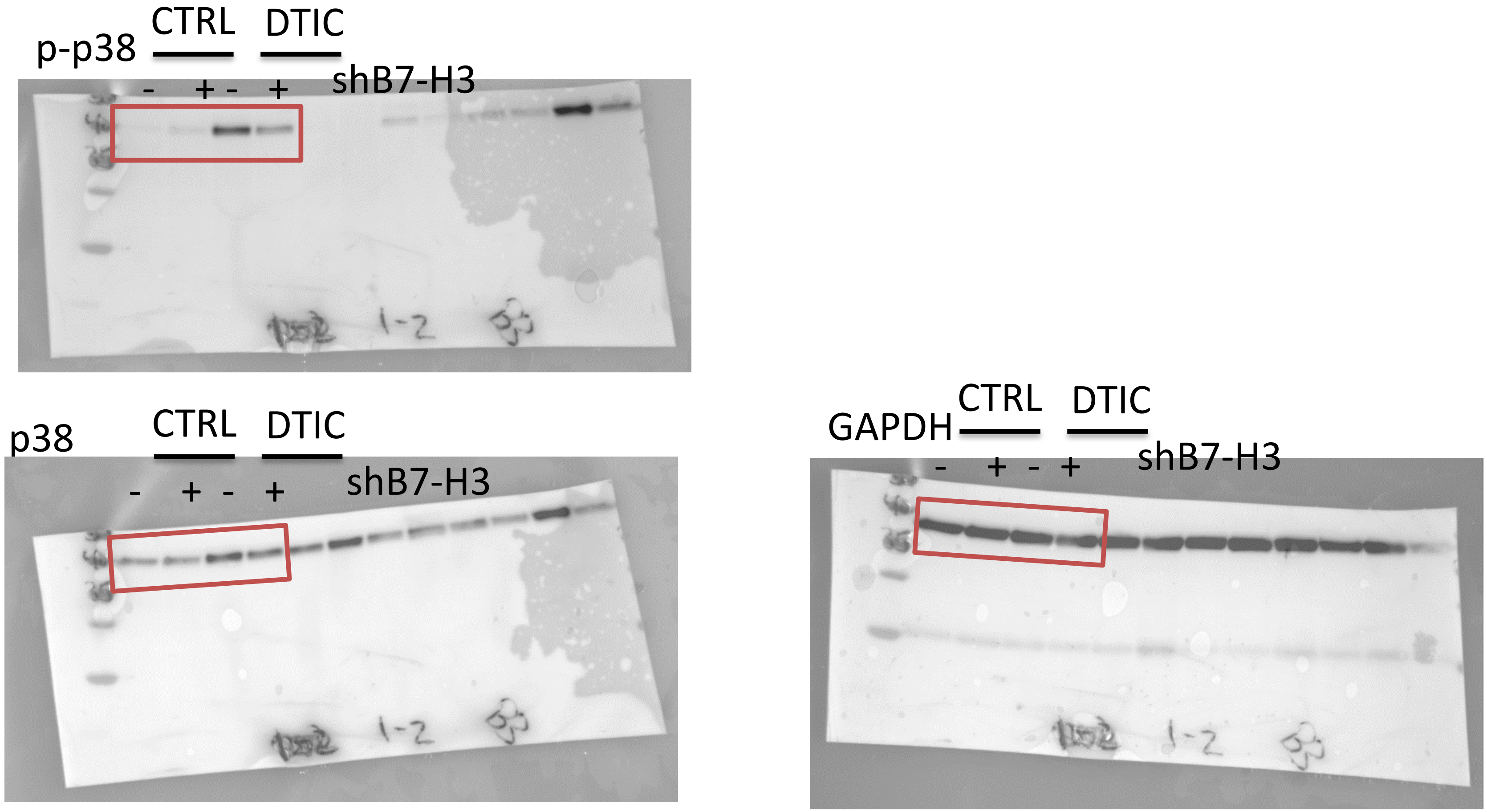
p-p38
CTRL
DTIC
- + - +
shB7-H3
p38
CTRL
DTIC
- + - +
shB7-H3
GAPDH
CTRL
DTIC
- + - +
shB7-H3

FIGURE 4C

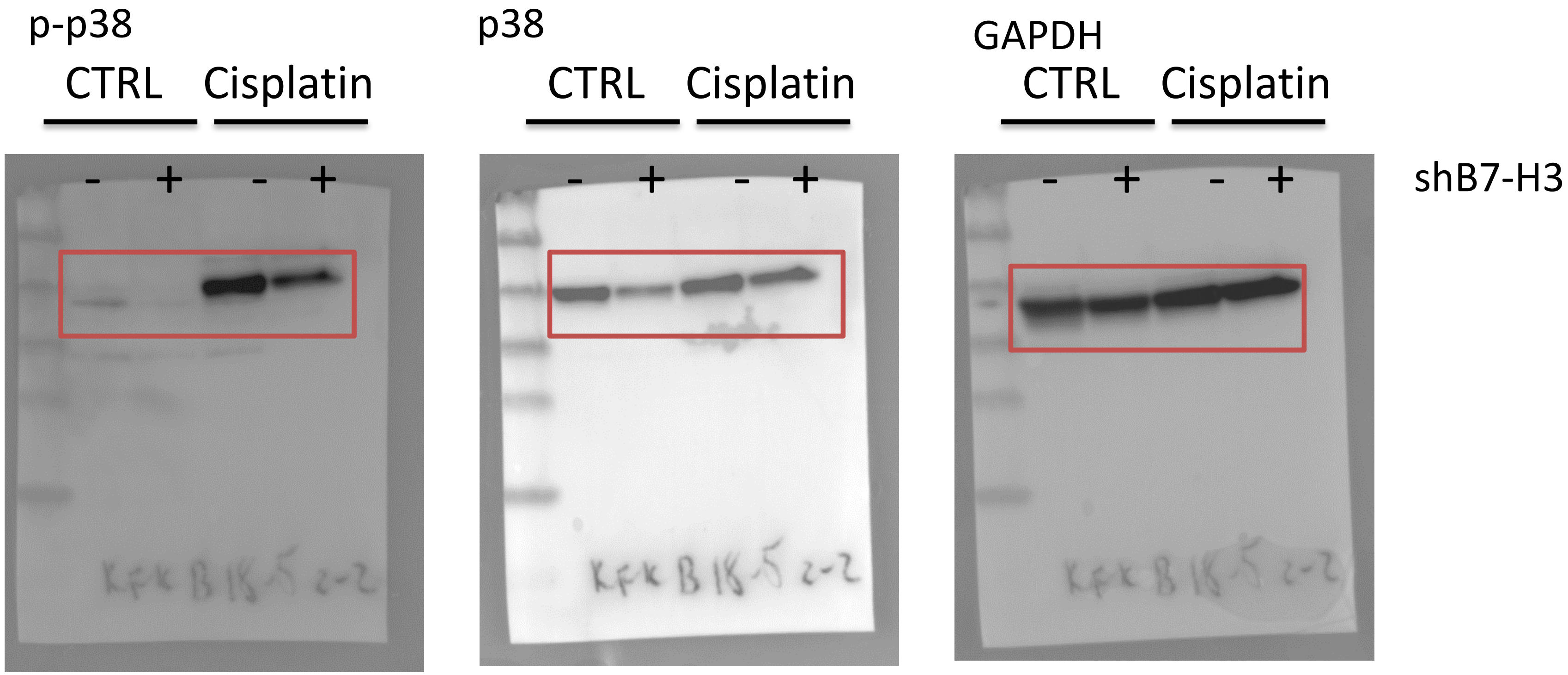
p-p38
CTRL
Cisplatin
p38
CTRL
Cisplatin
GAPDH
CTRL
Cisplatin
- + - +
- + - +
- + - +
shB7-H3

FIGURE 5A

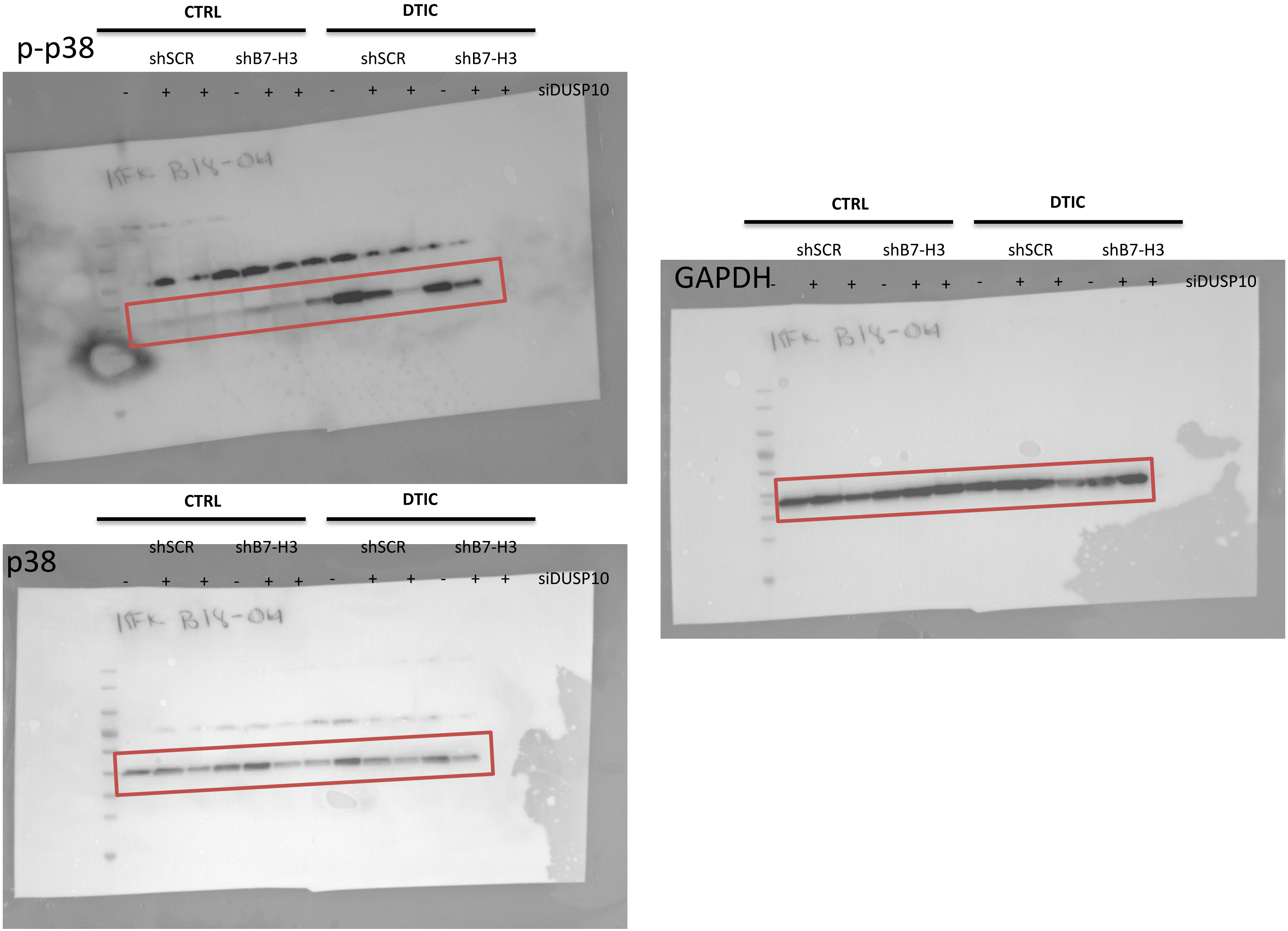
CTRL
DTIC
p-p38
shSCR
shB7-H3
shSCR
shB7-H3
- + + - + + - + + - + +
siDUSP10
CTRL
DTIC
p38
shSCR
shB7-H3
shSCR
shB7-H3
- + + - + + - + + - + +
siDUSP10
CTRL
DTIC
GAPDH
shSCR
shB7-H3
shSCR
shB7-H3
- + + - + + - + + - + +
siDUSP10
